# Rare Occurrence of Acute Hematogenous Periprosthetic Joint Infection Due to *Fusobacterium Nucleatum* in the Background of a Dental Procedure: A Case Report

**DOI:** 10.1111/os.12844

**Published:** 2020-11-04

**Authors:** Teng‐bin Shi, Xin‐yu Fang, Chao‐xin Wang, Yuan‐qing Cai, Wen‐bo Li, Wen‐ming Zhang

**Affiliations:** ^1^ Department of Orthopaedic Surgery First Affiliated Hospital of Fujian Medical University Fuzhou China

**Keywords:** Debridement, antibiotics, and implant retention (DAIR), *Fusobacterium nucleatum*, Periprosthetic joint infections

## Abstract

**Objective:**

*Fusobacterium nucleatum* is an anaerobic gram‐negative bacilli that is one of the oral and other mucosal surface microbiota. It involves a wide range of human diseases and was first found in periodontal diseases, but reports of bone‐related infections caused by *F. nucleatum* are rare, especially periprosthetic joint infections (PJI).

**Methods:**

Here, we present the first case of acute hematogenous PJI of the hip joint caused by *F. nucleatum*, and debridement, antibiotics, and implant retention (DAIR) was performed.

**Results:**

The patient was successfully treated with DAIR, identification of isolates by metagenomics next‐generation sequencing was confirmed by polymerase chain reaction.

**Conclusions:**

For stable acute hematogenous PJI after hip replacement, quick and accurate diagnosis, the identification of pathogenic microorganisms, and the use of DAIR combined with sufficient sensitive antibiotics have a certain clinical effect and can achieve the purpose of both preserving the prosthesis and infection control.

## Graphical abstract

Successful treatment of *F. nucleatum* PJI with DAIR (debridement, antibiotics, implant retention).

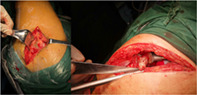

## Introduction

Periprosthetic joint infection (PJI) is a catastrophic complication after joint replacement. In any treatment for PJI, microbiological diagnosis is very important, and reliable clinical information about pathogenic microorganisms is crucial for the selection of appropriate treatment. At present, diagnosing PJI caused by rare microorganisms is still a challenge^1^.

*Fusobacterium nucleatum* is an anaerobic gram‐negative bacilli that is one of the oral and other mucosal surface microbiota. It involves a wide range of human diseases, was first found in periodontal diseases, and is one of the main pathogens of periodontitis^2^, but reports of bone‐related infections caused by *F. nucleatum* are rare, especially reports of PJI. Verma *et al*.^3^ reported the first case of periprosthetic infection of the hip caused by *F. nucleatum*, but the patient had sickle cell anemia. Corona *et al*.^4^ described the first case of acute periprosthetic joint infection of the knee caused by *F. nucleatum* in a non‐immunocompromised patient who was treated with a DAIR approach (debridement, antibiotics, and implant retention), which failed. There is still a lack of experience in the diagnosis and optimal treatment of PJI caused by *F. nucleatum* due to the lack of specific guidelines.

This paper reports the diagnosis and treatment of a patient with acute hematogenous PJI caused by *F. nucleatum* and discusses the difficulties surrounding pathogen diagnosis, surgical treatment, and antibiotic treatment for PJI in hope of increasing clinicians' knowledge of this bacterium.

## Case Report

**Fig. 1:**
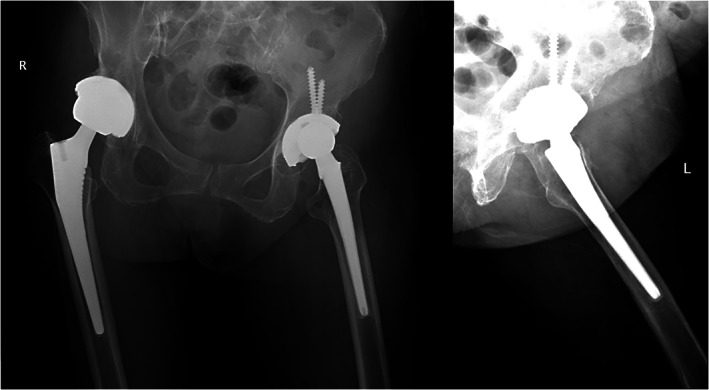
Admission X‐ray of the patient showed local bone resorption around the left prosthesis and no obvious loosening and dislocation of the left prosthesis.

A 71‐year‐old female was admitted to the hospital because of swelling and pain in the left hip. Fifteen years ago, she underwent cemented total hip arthroplasty because of left hip osteoarthritis. Five years ago, right total hip arthroplasty was performed for the same reason. Ten days ago, she developed progressive discomfort of the left hip incision that culminated in an episode of hip pain, fever (39.0 °C), chills, and local cutaneous changes. Then, she was transferred to our hospital. The patient had a history of a dental operation due to a “toothache” 1 month prior. The patient was diagnosed with diabetes 3 years ago and regularly took metformin and repaglinide. Pertinent physical findings on admission included a temperature of 36.8 °C, a pulse rate of 78/min, a respiratory rate of 22 breaths/min, and a blood pressure of 114/70 mm Hg. She had limpness and a high skin temperature of the left hip, an approximately 22‐cm surgical scar could be seen on the posterolateral side of both hips, no obvious exudation of the sinuses or pus was seen, and the left lower limb was approximately 5 cm shorter than the right lower limb. There was deep tenderness in the left groin, tapping tenderness in the left greater trochanter, and mild passive flexion of the left hip joint due to pain. Range of motion: abduction 40°, adduction 0°, flexion 70°, and hyperextension 0°. Laboratory analyses revealed a white blood cell count of 14.57 × 10^9^/L (88.8% neutrophils), a hemoglobin level of 12.6 g/dL, a C‐reactive protein (CRP, Beckman Coulter, Brea, California, USA) level of >90 mg/L, an erythrocyte sedimentation rate (ESR, Alifax, Genoa, Italy) of 58 mm/h, an albumin level of 29.0 g/L, and an HbA1c level of 6.2%. Tuberculosis antibody and T‐spot tests were negative. An admission X‐ray showed local bone resorption around the prosthesis and no obvious loosening or dislocation of the prosthesis (Fig. 1). Magnetic resonance imaging (MRI) of the left hip showed inflammatory changes in the upper part of the left thigh. Puncture of the left hip joint was performed under the guidance of ultrasound, but no specimens were obtained. However, according to the patient's history and symptoms, it was possible to diagnose acute hematogenous periprosthetic hip infection (the primary focus of oral infection), but the diagnosis still needed to be differentiated from the adverse reactions of metal debris around the prosthesis and polyethylene wear granule disease.

**Fig. 2:**
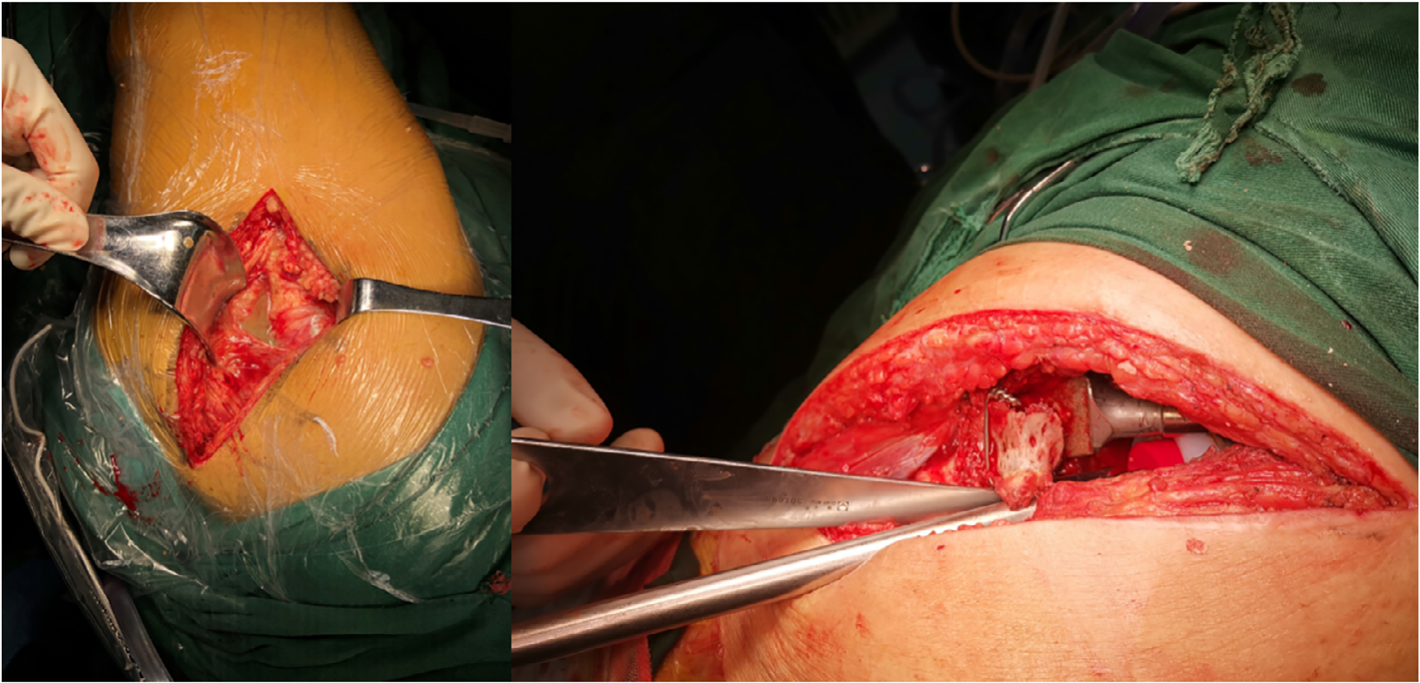
Intraoperative photograph shows a significant amount of thick dark pus and polyethylene abrasive particles in the subcutaneous and articular capsule.

At this point, a clinical diagnosis of left acute hematogenous periprosthetic hip infection was made, but the etiological diagnosis was not clear. We scheduled the patient for a debridement and implant retention (DAIR) procedure, according to

our institution's protocol for acute hematogenous periprosthetic joint infection. Before the operation, the patient received ceftriaxone intravenously for 5 days empirically. Under general anesthesia, we used the original posterolateral incision, the subcutaneous and articular capsules were found to be damaged, a significant amount of thick dark pus and polyethylene abrasive particles were found in the joint cavity (Fig. 2), and no obvious loosening of the femoral or acetabular prosthesis was found. The intraoperative synovial fluid was assessed for white blood cell count (WBC) and white blood cell differential was 176,959 × 10^6^/L, and the polymorphonuclear cell (PMN) count was 75%. The white blood cell counts of five intraoperative frozen sections of the periprosthetic tissues were all higher than 20/HPF. After removal of the polyethylene liner, pus and debris particles could be seen at the bottom of the joint cavity, accompanied by osteolysis. Pus and osteolysis could also be seen around the femoral prosthesis. Extended trochanteric osteotomy (ETO) was performed to expose the periprosthetic area of the femoral prosthesis. Pus was also seen in the medullary cavity. After complete radical debridement, the femur was fixed with a new steel wire, the acetabular side retained the original acetabular cup, and the new lining was fixed with bone cement. The joint was reduced, and the stability of the test was good. The synovial fluid, the periprosthetic tissue and the sonication fluid of the polyethylene liner were subjected to aerobic culture for 1 week and to anaerobic culture for 2 weeks. Five days after the operation, *F. nucleatum* was detected by metagenomics next‐generation sequencing (mNGS) and was confirmed by polymerase chain reaction (PCR) in the periprosthetic tissues. Seven days after the operation, *F. nucleatum* was isolated from two of five periprosthetic tissues, the sonication fluid of polyethylene liner with >100 colony forming units/plate. Thus far, a diagnosis of acute hematogenous PJI (the primary focus was oral infection) had been confirmed. After consultation with a microbiologist and an infectious disease doctor, we continued to use intravenous ceftriaxone and metronidazole for 2 weeks after the operation. After discharge, ceftriaxone combined with metronidazole was given intravenously for 10 days at a local hospital. Due to gastrointestinal reactions, metronidazole was stopped. The antibiotics were stopped 1 month later, and levofloxacin was taken orally sequentially for 6 weeks. The patient was followed regularly in the outpatient clinic after the operation. After 4 weeks of discontinuation, the CRP level and ESR were normal three consecutive times, and the incision had healed well, which was judged to represent infection remission.

**Fig. 3:**
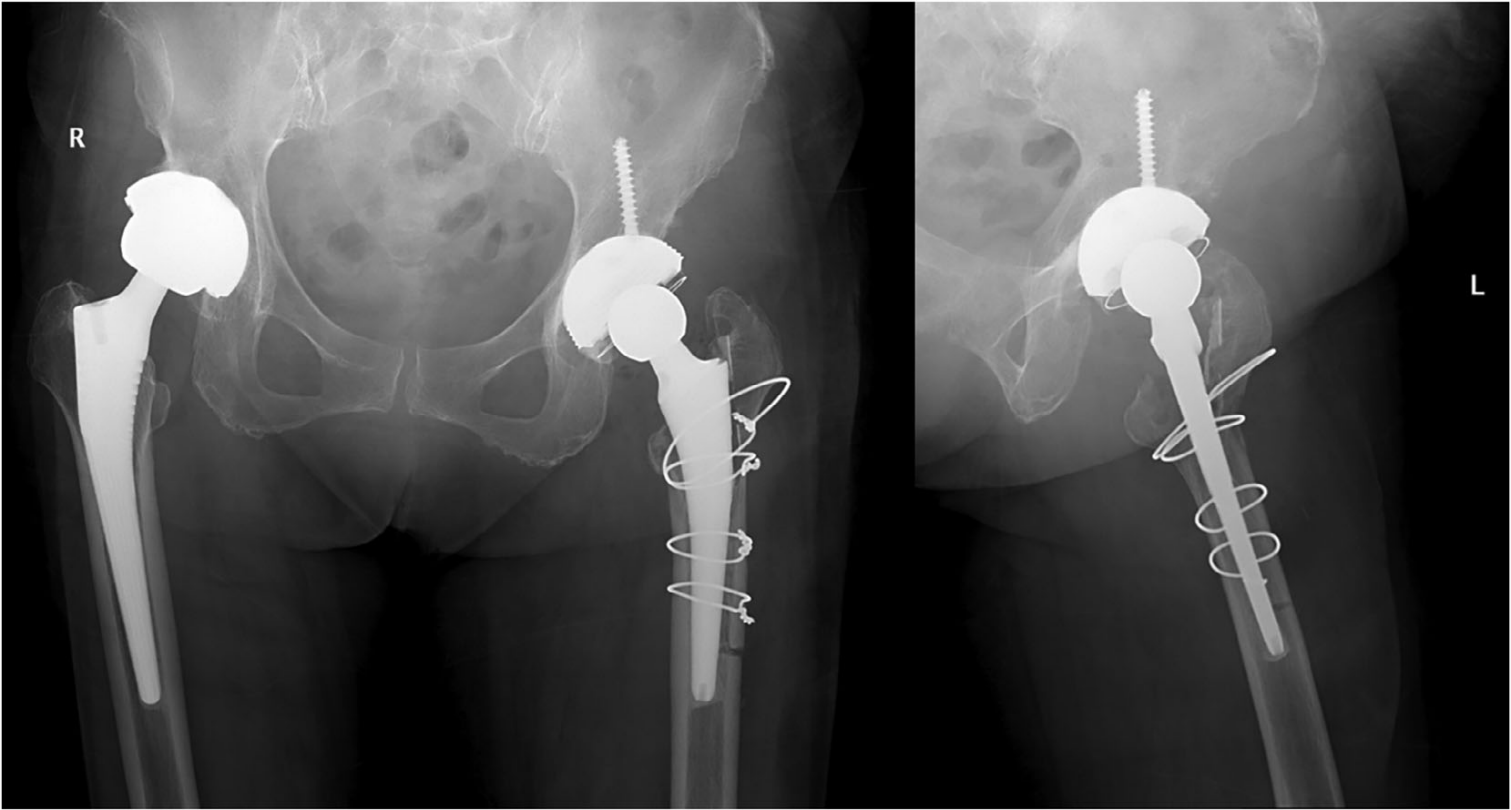
A postoperative follow‐up X‐ray showing the position of the prosthesis was good and the bone callus grew well, following eradication of *Fusobacterium nucleatum* infection.

After regular follow‐up for more than 1 year, the patient did not complain of left hip joint pain, the incision healed well, the patient could walk without crutches, and the patient recovered self‐care abilities. The CRP level and ESR were normal, and no other recurrence of infection was found. X‐ray and lateral radiographs of the left hip showed that the position of the prosthesis was good and that the callus had grown well (Fig. 3).

## Discussion

### *Infection of* F. nucleatum *Originating from Oral Cavity*

*Fusobacterium nucleatum* is a gram‐negative obligate anaerobic bacillus that is ubiquitous in the oral cavity. It is one of the main pathogens of periodontitis, pulpitis, and other inflammatory diseases. The adhesion and invasiveness of the bacteria are key to the mechanism of its colonization, transmission (local or blood origin), and evasion of the host response. Under normal circumstances, it is not found in other parts of the body or is rarely found, but it can cause extraoral infection in patients with underlying diseases^5^. At present, there are different reports on the mechanism of extraoral infection, including the spread of oral infection caused by transient bacteremia, causing the colonization of bacteria outside the oral cavity and then the release of free toxins and soluble antigen, causing systemic damage. Increasing evidence shows that oral symbionts and pathogens that spread to distant parts of the body translocate hematogenously and play an important role in infection and inflammation outside the oral cavity. Témoin *et al*.^6^ found bacterial DNA in the synovial fluid and that identical bacterial DNA was detected in the synovial fluid and dental plaque of patients with arthritis and periodontitis, suggesting that oral bacteria can translocate from the mouth to the synovial cavity.

We report a case of acute hematogenous PJI caused by *F. nucleatum*. The patient has no immunocompromised disease, but has a history of oral dental surgery 1 month ago, so it is possible to speculate the hematogenous spread from the patient's oral flora to the prosthesis. At present, the prophylactic use of antibiotic in patients with hip or knee replacement before dental operation is still controversial. Moreira *et al*.^7^ believe that there is no evidence to support or rule out the necessity of antibiotic prophylaxis as a means to reduce the risk of artificial joint infection in patients with periodontitis. Marculescu *et al*.^8^ suggest that the prophylactic use of antibiotics should be considered selectively in immunocompromised patients undergoing dental surgery.

A retrospective cohort study by Kao *et al*.^9^ found that there was no association between the incidence of PJI and prophylactic use of antibiotics, and that patients with hip or knee replacement had no increased risk of PJI after dental surgery and were not affected by antibiotic prophylaxis.

### *Diagnosis of Unusual PJI*

The early diagnosis of PJI and identification of pathogenic microorganisms are still the key to successful treatment; however, due to the limitations of culture technology, the culture time, the previous use of antibiotics, and other factors, the positive rate of bacterial culture is not satisfactory, the pathogenic microorganisms was unclear at the time of the second‐stage surgery in some patients, resulting in treatment failure^10^. In fact, etiological evidence cannot be found in approximately 7.0%–42.1% of patients with PJI during the entire treatment process^11^. In recent years, microbial molecular diagnostic techniques such as polymerase chain reaction (PCR), fluorescent *in situ* hybridization (FISH), and metagenomic next‐generation sequencing (mNGS) have been increasingly used in the diagnosis of PJI^12^, ^14^. In this case, the utilization of NGS and PCR contributes to diagnosis of PJI, it is suggested that the incidence of PJI caused by *F. nucleatum* and other caustic bacteria may be underestimated.

### *Treatment of Acute Hematogenous PJI*

At present, the main treatment methods for PJI are one‐stage revision, two‐stage revision, and debridement, antibiotics, and implant retention (DAIR). After comparing the risks of intraoperative bone loss and perioperative fracture, DAIR combined with sufficient antibiotics is recommended for the treatment of acute periprosthetic infection because of its advantages of a simple operation, short hospital stay, and low cost. In this case, DAIR was successfully used to treat acute periprosthetic hip infection; however, the choice for treatment of acute periprosthetic infection is still controversial^14^. Duque *et al*.^15^ describe a case of *F. nucleatum* prosthetic hip infection associated with a periprosthetic abscess in a woman with hemochromatosis who was successfully cured by two‐stage revision arthroplasty. A study by Wouthuyzen *et al*.^16^ suggests that DAIR seems to be a successful treatment strategy for early acute PJI, but for late acute PJI caused by *Staphylococcus*, the use of DAIR should be carefully considered. Due to the present lack of knowledge of *F. nucleatum*, which is a low‐incidence pathogen, further reporting should generate the knowledge needed to determine if the DAIR approach is appropriate for treatment.

The main limitation of this study is the short follow‐up time, and a long‐term follow‐up is needed. However, to the best of our knowledge, this is the first reported case of acute PJI of the hip joint caused by *F. nucleatum* that was successfully treated with debridement, antibiotics, and implant retention. For stable acute hematogenous PJI after hip replacement, quick and accurate diagnosis, the identification of pathogenic microorganisms, and the use of DAIR combined with sufficient sensitive antibiotics have a certain clinical effect and can achieve the purpose of both preserving the prosthesis and infection control.
